# Core psychiatry trainees views on MRCPsych course structure and delivery at East Midlands Deanery

**DOI:** 10.1192/bjo.2021.401

**Published:** 2021-06-18

**Authors:** Asma Javed, Hetal Acharya, Ian Yanson

**Affiliations:** 1 Leicestershire partnership NHS Trust; 2 Rampton Hospital

## Abstract

**Aims:**

The RCPsych curriculum for core training in Psychiatry (2013) requires each Deanery to run regional MRCPsych teaching programme.

The East Midlands School of Psychiatry run a local MRCPsych course aimed at all core psychiatry trainees in the deanery. Before the pandemic, the course took place between two venues – Nottingham and Leicester. During the pandemic, the course was delivered via Microsoft teams. We aimed to collect the feedback from trainees regarding the course to help shape the MRCPsych Course programme according to their training needs.

**Method:**

We devised an online Microsoft forms questionnaire which included:

Level of training

Number of exams passed

Relevance of MRCPsych content to clinical practice and membership exam

Usefulness of mock exams, simulation scenarios and workshops towards clinical and exam practice

Overall experience of the course

Which additional sessions they would like to be included

The effect of COVID-19 on their ability to attend in MRCPsych programme

These forms were sent to all the trainees in the region via email.

**Result:**

Out of 44 trainees, 9 responded. 66.6% of the trainees who responded were CT1 and 33.3% CT2. 45% had passed Paper A and 55% had not passed any exams. 78% of them agreed and 11% strongly agreed that course was relevant to the clinical practice. 55.6% agreed that course was relevant to membership course. 44.4% agreed and 11% strongly agreed that mock exams were useful. 66.7% agreed and 11% strongly agreed that simulation case scenarios and workshops were useful for exam and clinical practice. 22.2% strongly agreed and 33.3% agreed that sessions were engaging and motivating. Overall experience of MRCPsych exam was rated as excellent (11%), good (55%), satisfactory (22%) and poor (11%).

Suggestions to add additional sessions included antiracism in psychiatry, more mock exams, practical management of cases, to organise more interactive sessions on Microsoft teams, in-depth coverage of exam topics, to organise full day teaching sessions instead of half day.

33.3% of trainees commented that COVID-19 had impacted on their ability to attend the exam as initially face to face sessions were cancelled till end of May 2020 and when started there were technical issues with the online platform

**Conclusion:**

Consider feedback received in modifying aspects of the MRCPsych course

To share the results with trainers and course tutors

Arrange relevant mock exam sessions

Include the topics suggested by trainees and improve the experience of online learning by making it more interactive

Limitations: small sample size.

